# Dual-energy fluorescent x-ray computed tomography system with a pinhole design: Use of K-edge discontinuity for scatter correction

**DOI:** 10.1038/srep44143

**Published:** 2017-03-08

**Authors:** Tenta Sasaya, Naoki Sunaguchi, Thet- Thet-Lwin, Kazuyuki Hyodo, Tsutomu Zeniya, Tohoru Takeda, Tetsuya Yuasa

**Affiliations:** 1 Graduate School of Science and Engineering, Yamagata University, Japan; 2 Graduate School of Science and Technology, Gunma University, Japan; 3 School of Allied Health Sciences, Kitasato University, Japan; 4 Institute of Materials Structure Science, High Energy Accelerator Organization (KEK), Japan; 5 Graduate School of Science and Technology, Hirosaki University, Japan

## Abstract

We propose a pinhole-based fluorescent x-ray computed tomography (p-FXCT) system with a 2-D detector and volumetric beam that can suppress the quality deterioration caused by scatter components. In the corresponding p-FXCT technique, projections are acquired at individual incident energies just above and below the K-edge of the imaged trace element; then, reconstruction is performed based on the two sets of projections using a maximum likelihood expectation maximization algorithm that incorporates the scatter components. We constructed a p-FXCT imaging system and performed a preliminary experiment using a physical phantom and an I imaging agent. The proposed dual-energy p-FXCT improved the contrast-to-noise ratio by a factor of more than 2.5 compared to that attainable using mono-energetic p-FXCT for a 0.3 mg/ml I solution. We also imaged an excised rat’s liver infused with a Ba contrast agent to demonstrate the feasibility of imaging a biological sample.

X-ray fluorescence (XRF) analysis is a highly sensitive physicochemical method that enables the quantitative identification of an element of interest by collecting fluorescent x-ray photons emitted from the element. Synchrotron x-rays have excellent properties, such as high brilliances and collimation, and their use as sources enables the sensitivity of XRF to be increased further. Fluorescent x-ray computed tomography (FXCT) (also known as x-ray fluorescence computed tomography) using a synchrotron x-ray source has been developed based on the outstanding abilities of XRF. This technique can be utilized to delineate the spatial distributions of trace elements with high sensitivity, and it has many applications in materials and biomedical science^1^,^2^,^3^,^4^,^5^,^6^,^7^,^8^. In biomedical science, FXCT can potentially be used as a molecular imaging modality for small animals by delineating the distributions of trace elements within their organs. In preclinical studies, this capability enables the investigation of the causes, diagnosis, and treatment of diseases from a molecular perspective. Currently, other nuclear imaging modalities, such as positron emission tomography (PET) and single-photon emission computed tomography (SPECT) are also being developed for such purposes^9^,^10^,^11^. FXCT has an advantage over PET and SPECT in that it does not involve the use of a radioactive imaging agent. Recently, FXCT has also attracted attention as a strong candidate for the visualization of the distributions of gold nanoparticles^12^,^13^. Although FXCT has a wide range of uses, an imaging protocol that clearly provides the most efficient and quantitatively accurate detection of elements of interest has not yet been established.

The first image generated by performing FXCT using synchrotron x-rays was obtained from a physical phantom by utilizing imaging geometry based on first-generation computed tomography (CT). This imaging process involves acquiring a set of projections using a high-energy-resolution detector and a thin monochromatic parallel beam^14^,^15^ and has been employed to image the distributions of trace amounts of elements such as I in a variety of *ex vivo* and *in vivo* biological objects^16^,^17^,^18^. Although it enables highly sensitive detection with a spatial resolution of hundreds of micrometers, this approach is hindered by the long measurement time, which results from the projection data being acquired from translational and rotational scans of a thin pencil beam comparable in size to the resolution, with the beams being applied sequentially. Therefore, the FXCT images of biomedical samples reported thus far have been 2-D. For 3-D imaging, a scheme for the batch acquisition of 2-D projections is indispensable. Consequently, some batch acquisition methods for use in FXCT imaging have been proposed; in these techniques, projections are acquired from sheet-like or volumetric beams using detector arrays with long parallel collimators in front of the detective elements^19^,^20^. However, translational scans are still necessary in these methods, since the septa of the collimators produce regions in which detection is not possible.

We recently proposed a pinhole-based FXCT (p-FXCT) system with a 2-D detector and volumetric beam. This system enables faster data acquisition by completely eliminating the need for translational scans, and the first 3-D FXCT image of a physical phantom was obtained using this system^21^. However, the energy resolution of the 2-D detector was not sufficiently high to enable discrimination between the fluorescent and stray scattered x-rays according to their energies. Even if the detector was perpendicular to the incident beam so that photons scattered via Compton, Thomson, and multiple scattering could be prevented from impinging upon the detector, contamination was inevitable^22^. Since all of the detected x-ray photons were used in the reconstruction and were treated as if they were fluorescent x-ray photons, the background noise was enhanced, and the image quality deteriorated.

In this study, we focused on the photoelectric absorption discontinuity that occurs at the K-edges of trace elements. The fluorescent x-ray generation efficiency suddenly increases at an incident energy just above the K-edge in comparison with that just below the K-edge. In contrast, the Compton and Rayleigh scattering cross-section that contaminates the detected count scarcely changes between the incident energies just above and below the K-edge, since the energy difference is negligible. Therefore, by measuring the individual energies just above and below the K-edge, it may be possible to reduce the noise caused by scatter components effectively during the reconstruction process, although doing so would double the measurement time.

The purpose of this report is to propose and demonstrate a dual-energy p-FXCT imaging technique in which a low-energy-resolution 2-D detector is used and the K-edge discontinuities of trace elements are employed to correct for the deleterious effects of scatter components and improve the image quality. First, we describe the measurement process and introduce a statistical model to explain the corresponding reconstruction method, which is based on a maximum likelihood expectation maximization (ML-EM) algorithm. We then demonstrate the efficacy of the technique by describing a preliminary experiment in which a physical phantom and an I imaging agent were employed. Finally, we imaged an *ex vivo* rat’s liver, which included a Ba imaging agent, to confirm that the proposed method could be used to reconstruct a biomedical sample in 3-D.

## Method

### p-FXCT imaging setup and measurement process

**Figure 1:**
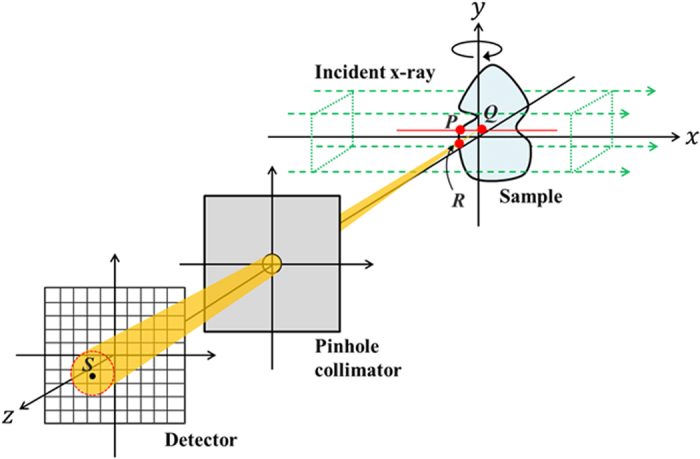
Schematic of the imaging geometry used for p-FXCT.

Figure 1 depicts the p-FXCT imaging geometry in the *xyz* coordinate system. A monochromatic and parallel incident beam propagates along the *x*-axis, and a pinhole collimator is set so that its surface is perpendicular to the *z*-axis and its center lies on the *z*-axis. The surface of a 2-D detector is set perpendicular to the *z*-axis. The incident volumetric beam irradiates an object set near the origin, with the volumetric beam covering the object. Imaging agents in the object, such as I, Gd, and Au, are excited by the incident beam and isotropically emit fluorescent x-ray photons upon de-excitation. Of all of the fluorescent x-ray photons emitted from all of the imaging agents in the object, only those passing through the pinhole are acquired by the detector. The projection acquisition process is repeated while rotating the object around the *y*-axis. In the following discussion, we assume that K_α1_ fluorescence is dominant, although other types of fluorescence, such as K_α2_ and K_β_ fluorescence, can easily be incorporated into the subsequent expressions, despite making the formulation more complicated.

Let us consider quantitatively how fluorescent x-ray photons are detected at an element on the detective surface. Consider a point *Q* inside the object during the flux of incident x-rays and a point *S* at the center of the detective element, as indicated in Fig. 1. We assume that *P, Q*, and *R* are the points of intersection between the incident flux and the object surface, between the incident flux and the line connecting *S* to the pinhole center, and between a line connecting *Q* to *S* and the object surface, respectively.

First, we will consider the measurement process when the above assumption holds. The process is divided into the following three subprocesses:

- attenuation of the incident beam by the object as the beam travels from *P* to *Q*;
- emission of fluorescent x-rays at *Q*; and
- attenuation of the fluorescent x-rays by the object as they travel from *Q* to *R*; the attenuated x-rays then travel to *S.*

In subprocess (a), the density of incident x-ray photons having reached *Q* is 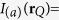 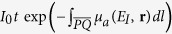 [photons/mm^2^] due to the attenuation that occurs as they travel along line segment 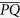, where **r**_*Q*_ represents the coordinates of *Q*; 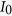
[photons/mm^2^/s] is the number of incident photons per unit area and time; 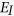 [keV] and 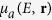 [1/mm] are the incident x-ray energy and the 3-D map of the total linear attenuation coefficient inside the object at incident energy *E*, respectively; and *t* [s] is the exposure time.

In subprocess (b), the quantity of fluorescent x-rays emitted at *Q* is proportional to the product of the incident photon density 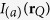 [photons/mm^2^] and the imaging element concentration 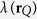 [g/mm^3^], which can be expressed as 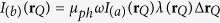
[photons], where *μ*_*ph*_ [mm^2^/g] and 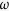 are the photoelectric mass absorption coefficient of the trace element and the fluorescent yield, respectively, and 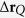
[mm^3^] is an infinitesimal volume in the vicinity of *Q*.

In subprocess (c), of all of the fluorescent x-ray fluxes emitted isotropically from *Q*, only the fluxes passing through the pinhole reach the detector, and the passing fluorescent fluxes are attenuated by the object. This attenuation is represented by the attenuation that occurs as the x-rays travel along line segment 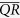, since the pinhole area is sufficiently small, although each flux is actually attenuated differently according to its propagation direction. Therefore, the number of fluorescent photons emitted from *Q* and reaching *S* is given by 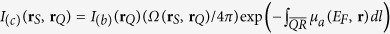
[photons], where 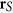 represents the coordinates of *S*, and 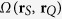 [steradians] and *E*_*F*_ [keV] are the solid angle subtended by *Q* through the pinhole toward the detective element centered at *S* and the fluorescent x-ray energy, respectively.

The amounts of attenuation that occur along 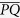 and 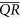 are unknown unless tomographic reconstruction is performed. In this research, we assumed that the maps of the attenuation coefficients at the incident and fluorescent energies, *i.e*., 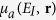 and 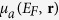, respectively, were known in advance. To acquire the maps, it is necessary to perform two separate CT reconstructions. However, acquiring two extra sets of CT measurements would increase the measurement time and exposure dose. Actually, it is possible to approximate the region in which the sample exists as a homogeneous water region. If this approximation is employed, the theoretical values of the attenuation coefficients at the incident and fluorescent energies are known. Such approximations are also widely performed in attenuation correction in PET and SPECT. Another option is to incorporate attenuation coefficient estimation into the reconstruction process. This option is very challenging, and we would like to investigate it further in future work.

In the subsequent discussion, the number of fluorescent x-ray photons that are counted at the detective element centered at **r**_s_ is denoted as


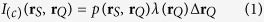


where


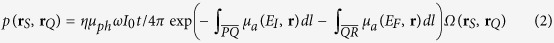


and *η* is the quantum efficiency of the detector. If the imaging geometry and coordinates of *S* and *Q*, 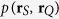 are known in advance, because the maps of 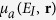 and 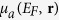 can be measured by performing ordinary CT with monochromatic x-rays.

The above discussion concerns the contribution of fluorescent x-rays from *Q* to the count at *S*, although the contributions from all of the regions around *Q* should actually be considered. Therefore, the total measured count at *S* is given by


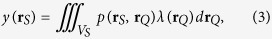


where *V*_*S*_ is a region in the object inside a cone subtended by *S* toward the pinhole.

**Figure 2:**
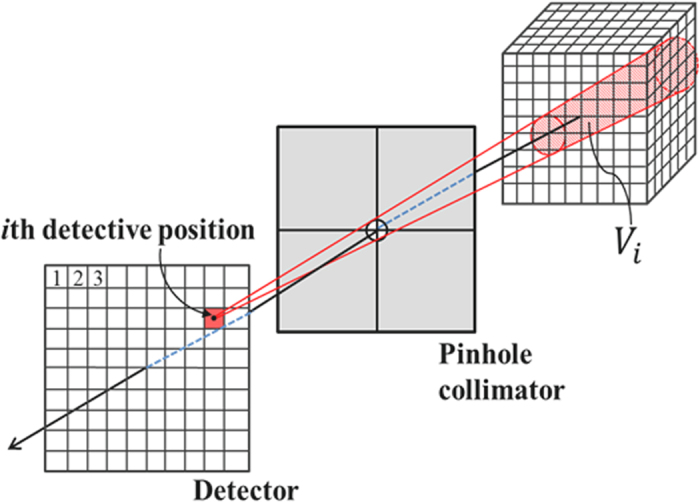
Discretization for reconstruction: *V*_*i*_ is an object region inside the cone subtended by the *i*th voxel towards the pinhole. The *i*th detective position collects all of the fluorescent photons from the voxels in cone *V*_*i*_.

To perform reconstruction, the object is discretized into voxels as depicted in Fig. 2. Here we assume that the object is fixed to the *xyz* coordinate system during the projection acquisition process and that the incident beam, pinhole collimator, and detective surface are rotated around the *y*-axis, with their positional relationship maintained, although the object is rotated when actual measurements are performed. We introduce the index *j* (=1, 2, …, *N*) to identify the voxel in the lexicographic ordering corresponding to **r**_*Q*_, where *N* is the number of voxels. Additionally, we introduce the index *i* (=1, 2, …, *M*) to denote the positions of the individual detective elements corresponding to 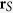 throughout the projection acquisition process, where the positions of the detective elements are numbered consecutively, and *M* is the number of positions. As a result, Eq. (3) can be discretized to


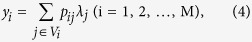


where *y*_*i*_, *p*_*ij*_, and λ_*j*_ correspond to 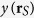, 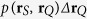, and 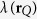, respectively; *y*_*i*_ and *p*_*ij*_ are known; and λ_*j*_ requires estimation.

In the above discussion, only the fluorescent x-ray photons were considered, whereas in reality, the scattered x-rays are counted at each detective position simultaneously. Thus, Eq. (4) can be modified to


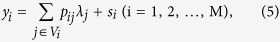


where *s*_*i*_ denotes the count of scattered x-rays contaminating the count at the *i*th detective position and includes all of the contributions caused by Compton, Rayleigh, and multiple scattering, other than fluorescent x-rays.

### Dual-energy p-FXCT

The use of a high-energy-resolution detector, such as the solid-state detectors used in first-generation FXCT, can enable the fluorescent counts to be differentiated from the measured counts contaminated with scattered counts^14^,^15^. However, in our situation, it is impossible to identify fluorescent x-rays as the only signal components. We therefore focused on the K-edge. Generally, each element has a different K-edge, which is the binding energy of the K-shell electron of an atom of that element. The photoelectric absorption coefficient *μ*_*ph*_ behaves discontinuously at the K-edge; that is, it suddenly increases at incident energies just above the K-edge, whereas it monotonically decreases at energies below the K-edge. This phenomenon has long been used in radiology to increase the contrasts of lesions including imaging agents, by subtracting images acquired just below the K-edge from those obtained just above the K-edge. This method is known as K-edge subtraction^23^.

Based on Eq. (5), the number of fluorescent photons just above the K-edge can be expected to be much higher than that just below the K-edge, since *p*_*ij*_ includes *μ*_*ph*_. However, the scatter component *s*_*i*_ that originates from Compton and Rayleigh scattering depends on the incident energy. The scattering parameters have similar values just above and below the K-edge, since the difference between the high and low incident energies is very small. Therefore, the scatter components can be eliminated using the two sets of projections acquired at individual incident energies just above and below the K-edge. We assume that the two scanning sets are described by the following equations:


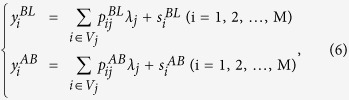


where 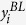 and 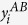 are the counts measured at energies just below and above the K-edge, respectively; 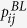 and 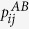 are the coefficients corresponding to 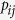 at energies just below and above the K-edge, respectively; and 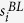 and 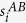 are the scatter components at energies just below and above the K-edge, respectively.

Simple subtraction, such as that involved in the K-edge subtraction method, would only enhance the noise, since the signal-to-noise ratios (S/N) of the projections are low. We therefore decided to utilize the ML-EM algorithm, which is widely used in PET and SPECT, to enable satisfactory reconstructions to be obtained from low-S/N projections^24^,^25^.

### Reconstruction based on the ML-EM algorithm

The key component of scatter contamination suppression is making the probabilistic properties of the scatter components above and below the K-edge equivalent to each other, *i.e.*, to render the means of the Poisson distributions specifying both scatter components equivalent to each other, instead of directly making their counts equivalent or performing K-edge subtraction. To derive a reconstruction scheme based on the ML-EM algorithm, we first introduced the following statistical model:


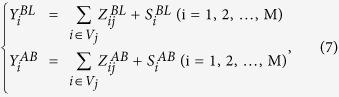


where 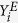, 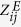, and 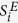 (*E *=* BL, AB*) are random variables representing the total count measured at the *i*th detective element position, the count of fluorescent x-rays emitted from the *j*th voxel and detected at the *i*th position, and the count of scattered x-rays detected at the *i*th position, respectively. These quantities are assumed to be independent according to the following Poisson distributions:


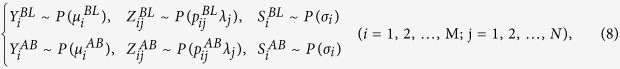


where *P(α*) is a Poisson distribution with a mean of *α*, and 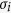 is the mean of the random variables 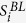 and 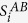 (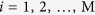). From Eqs (7) and (8), it can be observed that the following relations hold:


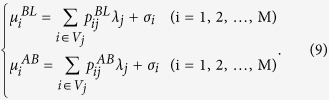


Here, it is assumed that 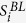 and 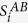 obey identical Poisson distributions because the energy difference between below and above the K-edge is very small, which is the key to the proposed reconstruction algorithm.

Letting the complete data consist of 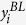, 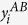, 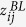, 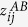, 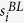, and 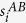 (*i* = 1, 2, …, *M*; *j* = 1, 2, …, *N*), where the last four values are unobservable, the complete-data likelihood is


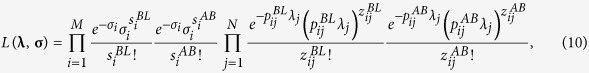


and the complete-data log likelihood is


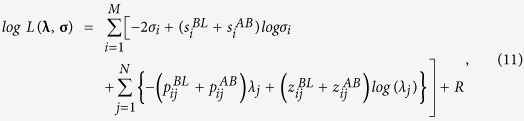


where 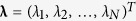, 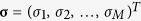, and *R* is a term irrelevant to the following calculation.

In step *E* of the (*n* + 1)th iteration, it is necessary to calculate the conditional expectations of 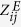 and 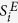 (*E *=* BL, AB*) given the observed data 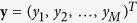, using the values 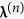 and 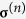 that are obtained after the *n*th iteration for **λ** and **σ**. From calculations based on Bayes’ theorem, the conditional distributions of 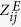 and 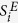 (*E* = *BL, AB*) are determined to be binomial with sample size parameter *y*_*i*_ and the respective probability parameters


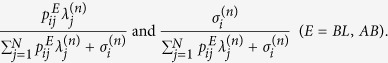


Therefore, the conditional expectations of 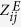 and 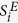 (*E* = *BL, AB*) are


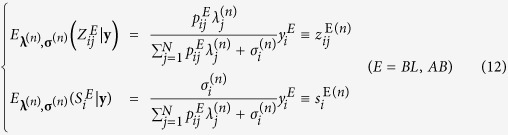


In step *M*, 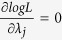 and 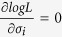 are solved with respect to *λ*_*j*_ and *σ*_*i*_, respectively, and 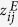 and 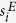 are then replaced with 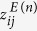 and 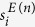 (*E = BL, AB*) using Eq. (12). Finally, the estimators in the (*n* + 1)th iteration can be obtained as follows:


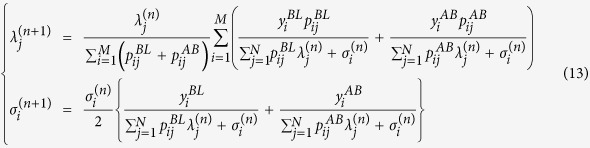


for each *i* (=1, 2, …, *M*) and *j* (=1, 2, …, *N*).

## Experiment

**Figure 3:**
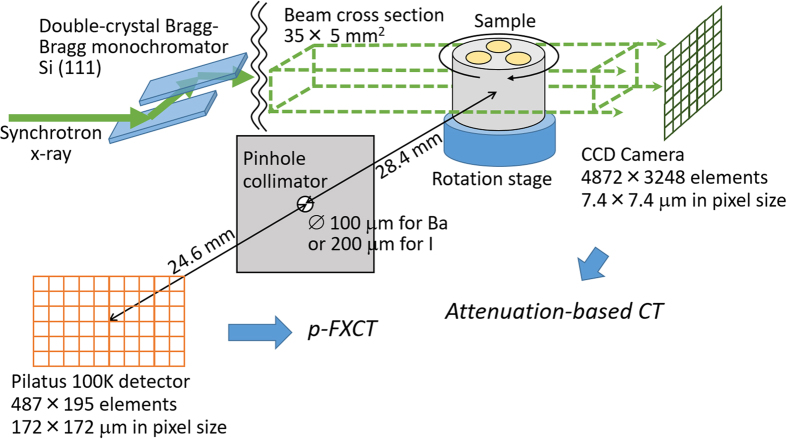
Experimental parameters.

We constructed a p-FXCT imaging system using the bending-magnet beamline AR-NE7A (6.5 GeV) at KEK. The imaging system consisted of a double-crystal Bragg–Bragg monochromator using Si(111) single crystals, an x-ray shutter and slit system, a rotational stage for object positioning, a 500-μm-thick W plate with a pinhole, and a Pilatus 100 K detector with 487 × 195 elements (pixel size : 172 × 172 μm^2^), whose energy resolution was not sufficiently high to enable discrimination of the fluorescent x-rays at the incident energy of around 30 keV. This detector was selected despite its insufficient sensitivity in energy regions around 30 keV because it yields high-S/N measurements since there is no dark current and also offers a data transmission rate higher than those of other types of 2-D detectors, such as charge-coupled device (CCD)-based detectors. The cross-section of the incident beam was collimated to 35 mm (horizontal) ×5 mm (vertical) by the slit. The distances between the center of the rotational stage and the pinhole collimator and between the pinhole collimator and the detective surface were 28.4 mm and 24.6 mm, respectively (Fig. 3). The rotational stage and detector were controlled by a PC.

**Figure 4:**
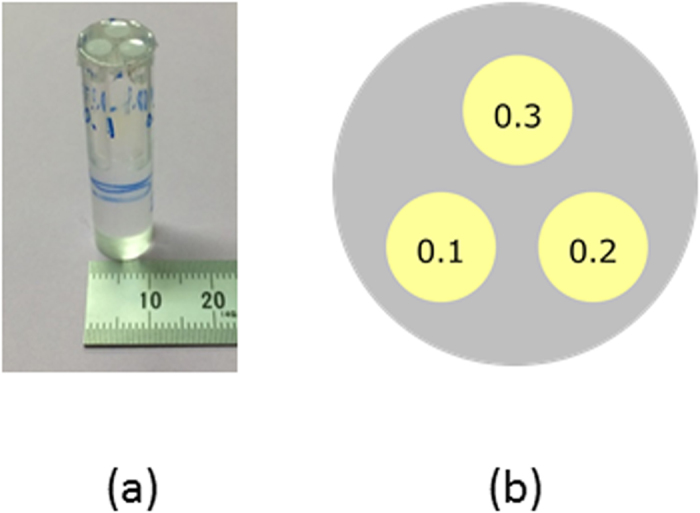
Physical phantom used for image contrast improvement evaluation. It consists of a 10-mm-diameter acrylic cylinder with seven 3-mm-diameter channels filled with different concentrations of I solution (0.1, 0.2, 0.3 mg/ml): (**a**) a photograph of the whole phantom and (**b**) cross-section of the phantom and arrangement of I concentrations.

A physical phantom was prepared to evaluate the contrast-to-noise ratio (CNR). This phantom consisted of a 10-mm-diameter acrylic cylinder with three 3-mm-diameter channels filled with different concentrations of I solution (0.1 mg/ml, 0.2 mg/ml, and 0.3 mg/ml), as shown in Fig. 4. The pinhole diameter was 0.2 mm, and the distances between the rotational axis and the pinhole plane and between the pinhole plane and the detective surface were 27.4 mm and 32.5 mm, respectively. The incident energies just above and below the K-edge of I were tuned to 33.4 keV and 33.0 keV, respectively. The photon flux rate in front of the object was about 5.0 × 10^8^ photons/mm^2^/s at energies near the K-edge of I (33.2 keV) for a storage-ring current of 40 mA. The exposure time for a single projection was 1.0 min. A total of 120 projections were obtained in 3° intervals covering a 360° range. To calculate *p*_*ij*_, the linear total attenuation coefficients of water for the 33.4 keV and 33.0 keV incident beams and 28.3 keV fluorescent beams were set to 0.0316 mm^−1^ and 0.0385 mm^−1^, respectively. Since the I solution was diluted and the linear attenuation coefficients of acrylic are close to those of water, we regarded the object as homogeneous for the attenuation part of the process. The other parameters included *μ*_*ph*_ = 3.51 × 10^3^ mm^2^/g at 33.4 keV and 0.58 × 10^3^ mm^2^/g at 33.0 keV, *ω* = 0.88, and voxel dimensions of 172 × 172 × 172 μm^3 ^26.

**Figure 5:**
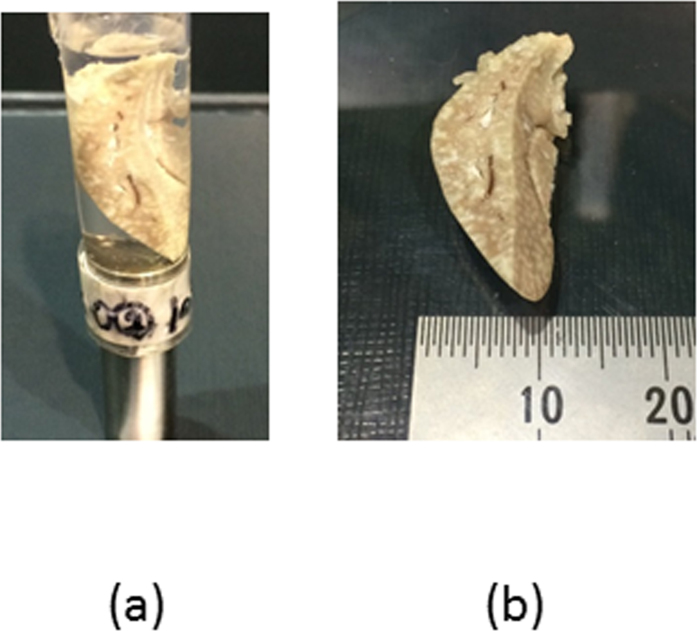
Excised rat’s liver: (**a**) the sample in a formalin-filled container and (**b**) the sample itself.

We imaged an *ex vivo* liver from a normal rat to demonstrate the suitability of this technique for biomedical imaging. A 7-week-old male Wistar rat with a body weight of 300 g was anesthetized via an intraperitoneal injection of sodium pentobarbital (50 mg/kg body weight). The apex of the left ventricle was surgically cannulated, and the rat’s blood was replaced with 500 ml of barium sulfate solution (200 mg/ml), which contained heparin to eliminate blood coagulation artifacts within the blood vessels. Immediately after the perfusion, the hepatic artery and hepatic vein were ligated, and the liver was extracted. The liver was then fixed in 10% formalin for imaging, as shown in Fig. 5. The experimental protocol was approved by the President of Kitasato University through the judgment of the Animal Care and Use Committee of Kitasato University (approval no. 1502). All of the processes were performed in accordance with the guidelines and regulations of the American Physiological Society. A pinhole diameter of 0.1 mm was used, and the distances between the rotational axis and the pinhole plane and between the pinhole plane and the detective surface were set at 28.0 mm and 32.5 mm, respectively. The incident energies just above and below the K-edge of Ba were tuned to 37.6 keV and 37.2 keV, respectively. The photon flux in front of the object was almost identical to that at the K-edge of I. The exposure time for a single projection was 20 s. A total of 180 projections were acquired in 2° intervals covering a 360° range. To calculate *p*_*ij*_, the linear total attenuation coefficients of water for the 37.6 keV and 37.2 keV incident beams and 31.8 keV fluorescent beams were set to 0.0245 mm^−1^ and 0.0282 mm^−1^, respectively; *μ*_*ph*_ was 2.85 × 10^3^ mm^2^/g at 37.6 keV and 0.483 × 10^3^ mm^2^/g at 37.2 keV, and *ω* was 0.90^26^. The size of each voxel was 172 × 172 × 172 μm^3^. For the purposes of comparison, ordinary tomographic images based on attenuation contrast were acquired using a CCD camera (X-ray FDI VHR 16 M manufactured by Photonics Science) with a pixel size of 7.4 × 7.4 μm^2^ and an image array size of 4872 × 3248. The camera was positioned downstream of the sample and used with 2 × 2 on-chip binning. The incident energy was 37.6 keV, the flux rate was 1.5 × 10^7^ photons/mm^2^/s, the distance between the rotational axis and the detective surface was 60 cm, the exposure time was 120 ms, and a total of 360 projections were acquired in 0.5° intervals covering a 180° range.

The resolutions of images reconstructed using the proposed method can be improved by reducing the pinhole diameter to limit the directions from which the fluorescent photons can originate. However, the number of fluorescent photons reaching the detector simultaneously decreases. Consequently, the measurement time must be extended to collect a sufficient number of photons to achieve the same S/N. Thus, there is a trade-off relationship between these parameters. Since our target resolution was about 0.3 mm and the *in vivo* measurement time should be less than 30 min, which is the time for which the anesthetic is effective, we empirically adopted 0.2- and 0.1-mm-diameter pinholes for the physical phantom and the rat’s liver sample, respectively. The proposed reconstruction algorithm was implemented using C# on a PC with Windows 8, Intel Xeon CPU E5-2620, and 192 GB of memory. The voxel size was 70 × 70 × 40, the number of expectation maximization (EM) iterations was 20, and the total calculation time was about 30 min.

## Results

### Physical phantom

**Figure 6:**
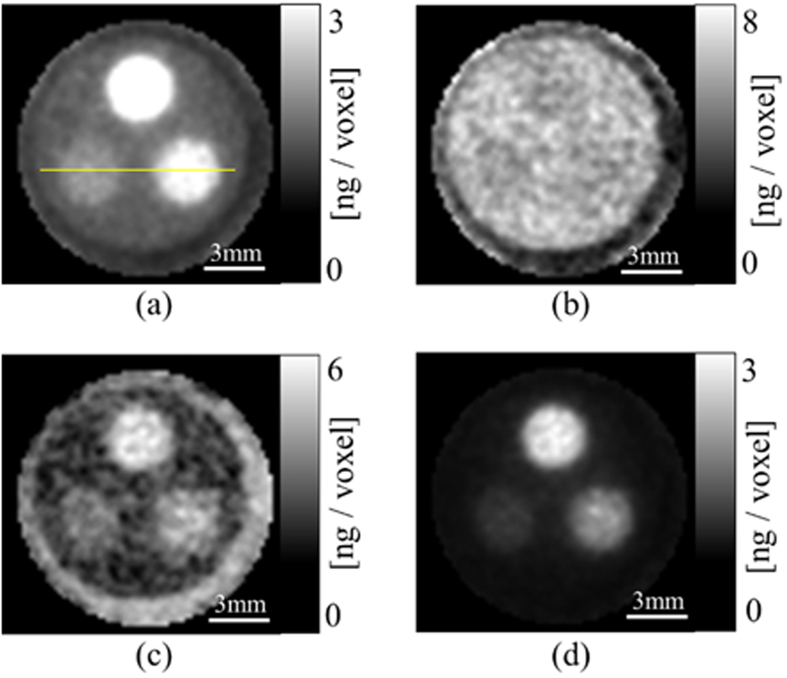
Slices at the central level of the 3-D images reconstructed by (**a**) mono-energy imaging just above the K-edge of I, (**b**) mono-energy imaging just below the K-edge, (**c**) subtracting the image in (**b**) from the image in (**a**), and (**d**) dual-energy imaging.

**Figure 7:**
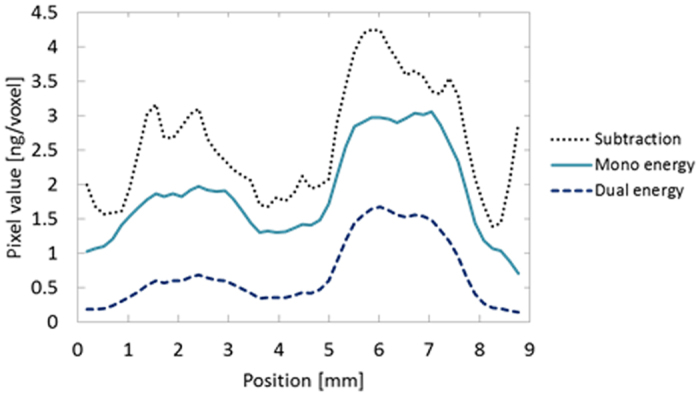
Profiles of voxel values along the yellow line in Fig. 5(a).

Figure 6 presents slices from the central levels of the 3-D reconstructed images of the phantom. Figures 6(a) and (b) were reconstructed from the projections obtained at incident energies just above and below the K-edge of I using the conventional ML-EM algorithm without incorporating a scattering process, Fig. 6(c) is the difference obtained by subtracting Fig. 6(b) from Fig. 6(a) and (d) is the image obtained from the proposed dual-energy imaging process. Each image has dimensions of 80 × 80 pixels. In Fig. 6(a), the regions in which I is not present have pixel values higher than those in Fig. 6(d). Figure 6(b) exhibits little contrast anywhere, since there is very little fluorescence emission. In Fig. 6(c), noise caused by scatter components is enhanced since this image was obtained by simple subtraction. Figure 6(d) demonstrates that the background noise caused by scatter components was effectively suppressed by performing dual-energy FXCT. Figure 7 depicts the profiles of dual-energy, mono-energy, and subtraction FXCT images obtained along the yellow line in Fig. 6(a). Comparison of the profiles in Fig. 7 demonstrates that the background regions in which I is not present are effectively suppressed by dual-energy FXCT.

Figure 6(b) indicates that the I-containing regions are not delineated in the image below the K-edge, which demonstrates the existence of photoelectric absorption discontinuity at the K-edge. The subtraction image in Fig. 6(c) does not clearly depict the I-containing regions, suggesting that performing subtraction without incorporating a scattering process into the measurement model increases the noise. From Fig. 6(d), it can be subjectively determined that dual-energy reconstruction satisfactorily suppresses the signal in the acrylic regions. In Fig. 6(a), non-negligible signals can be observed even in the acrylic regions, as in mono-energy imaging just above the K-edge, since the scatter components are treated as fluorescent components.

**Figure 8:**
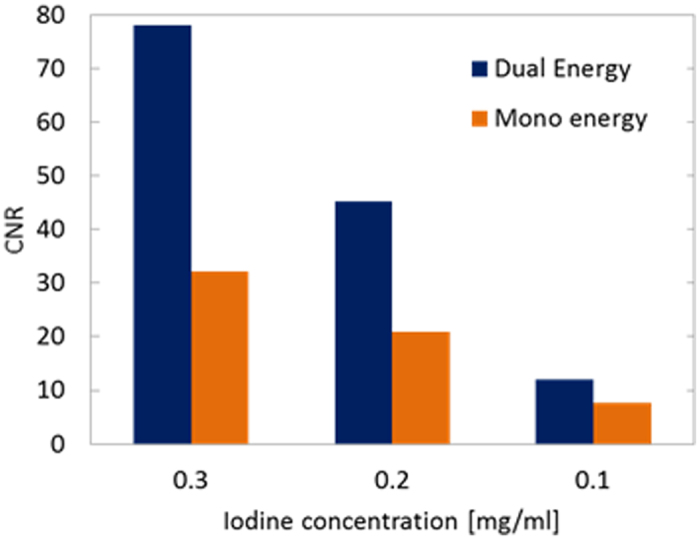
Comparison of the CNRs in dual- and mono-energy imaging.

To compare the image contrast between the mono- and dual-energy reconstructions, we measured the CNRs of the images in Fig. 6(a) and (d). The CNR is defined as


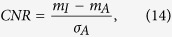


where *m*_I_ and *m*_A_ the means of the 1 × 1 × 1 mm^3^ voxel values in the I and acrylic regions, respectively, of the central slice image, and *σ*_*A*_ is the standard deviation in the acrylic region. The CNR was calculated for each concentration of I for the images obtained by performing mono- and dual-energy imaging. The results are presented in Fig. 8, and it is evident that the CNR is greater in the dual-energy results than in the mono-energy results at all of the concentrations examined. This improvement is especially evident in the CNR in the 0.3 mg/ml region, which is more than 2.5 times higher in the dual-energy imaging case than in the mono-energy imaging case. In the 0.1 mg/ml region, the CNR remains 1.3 times higher in the dual-energy imaging case.

These results indicate that the proposed dual-energy imaging method enables effective delineation of trace quantities of imaging elements.

### Blood vessels in the rat liver

**Figure 9:**
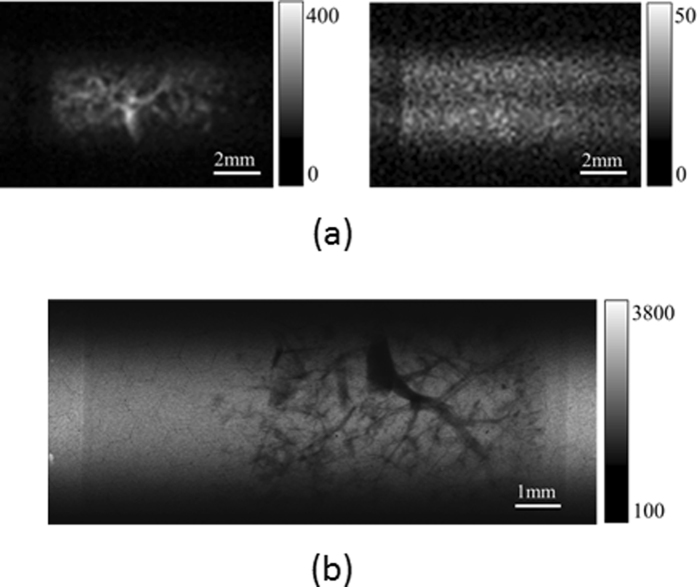
Example projections of blood vessels in an excised rat liver: (**a**) p-FXCT (left: above K-edge, right: below K-edge) and (**b**) attenuation-based CT.

**Figure 10:**
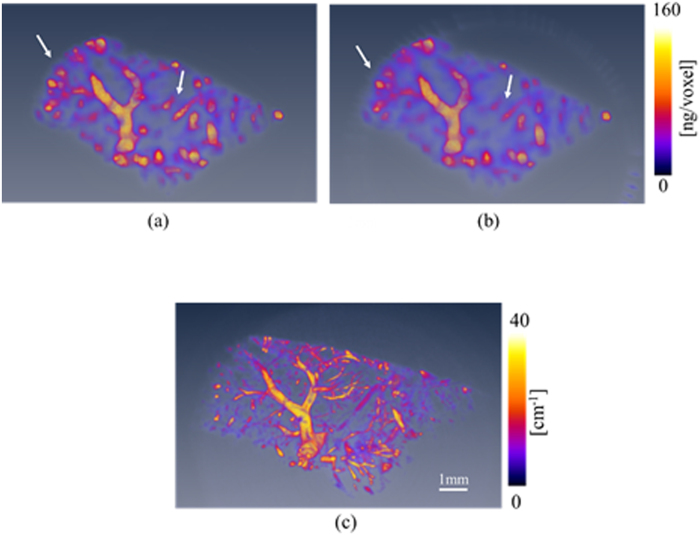
Volume renderings of blood vessels in the rat’s liver obtained using (**a**) dual-energy FXCT (see Visualization 1), (**b**) mono-energy FXCT (see Visualization 2), and (**c**) attenuation-contrast CT (see Visualization 3).

Examples of the projections obtained by performing p-FXCT and attenuation-based CT are presented in Fig. 9(a) and (b), respectively. The left and right images in Fig. 9(a) are the projections acquired by conducting p-FXCT just above and below the K-edge of Ba, respectively. The blood vessels in the p-FXCT projection obtained above the K-edge are recognizable in the right image in Fig. 9(a), although they are blurred. They are, however, clearly delineated in the projection obtained by conducting attenuation-based CT, which is shown in Fig. 9(b). Figure 10 presents 3-D renderings of the blood vessels in the rat liver. Figure 10(a) and (c) were reconstructed using dual-energy p-FXCT, mono-energy p-FXCT, and attenuation-contrast CT, respectively; notably, Fig. 10(a) and (b) are the world’s first 3-D FXCT images of a biological sample. The resolution of FXCT based on the pinhole effect is mainly limited by the pixel size. Since the pixel size was 172 × 172 μm^2^ in this study, the structures of blood vessels with the diameter less than 172 μm could not be imaged. On the other hand, the resolution of attenuation-based CT is mainly limited by the size of the detector pixels (7.4 × 7.4 μm^2^ in this investigation). Therefore, the resolution of the FXCT results is significantly inferior that of the attenuation-based CT results. However, the results obtained using these two methods are similar to each other on a larger scale. In the mono-energy FXCT image in Fig. 10(b), thin and fine-structured vessels are not sufficiently depicted in some regions (indicated by white arrows), unlike in the dual-energy FXCT image in Fig. 10(a). This difference is evident because the proposed dual-energy method improves the image contrast.

## Discussion

### Efficacy and limitations of dual-energy reconstruction

To perform the proposed reconstruction technique, we first prepared the extra random variables representing the scatter components below and above the K-edge, which are denoted as 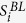 and 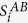, respectively. Then, to confine the counts due to different types of scattering into 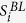 and 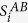, we assumed that both quantities obeyed Poisson distributions with a common mean because the difference between the energies below and above the K-edge is almost negligible. The significant contrast improvement evident in Fig. 6 is ascribable to the validity of the above assumptions.

Although using the proposed reconstruction method satisfactorily improved the contrast, the spatial resolution did not seem to be improved, as evidenced by Figs 6 and 7, since the super-resolution scheme was not incorporated into the proposed reconstruction algorithm. In other words, we set the voxel size to 172 × 172 × 172 μm^3^ in the present research, which was the same as the size of each pixel in each detector element. If the voxel size was reduced, the resolution could be improved although the amount of calculation required would simultaneously increase.

Since the main purpose of this research was to investigate the efficacy of the proposed method for scatter suppression, we did not optimize the calculation speed. In this study, we adopted the traditional ML-EM algorithm to solve the proposed reconstruction problem. Thus, the convergence rate was relatively low. If the ordered set expectation maximization (OSEM) method widely used in PET and SPECT were utilized instead, the convergence rate could be improved. Therefore, we are currently in the process of implementing OSEM for dual-energy FXCT.

In the proposed reconstruction method, we updated *λ*_*j*_ by using the data below and above the K-edge simultaneously, as described in Eq. (13). Another option would be to adopt an alternating update method. That is, *λ*_*j*_ could be updated by using the data below and above the K-edge in an alternating manner. The alternating method could reduce the amount of memory required by half. We plan to implement this method in the future to investigate whether the convergence rate could be improved or not.

### Further measurement time reduction

In this study, the experimental results demonstrated that the proposed dual-energy p-FXCT method suppressed scatter and improved the CNR. However, there is a practical limitation in that the exposure time of 2 min (1 min each above and below the K-edge) for acquisition of a single projection in the phantom-imaging experiment is too long for *in vivo* imaging applications. However, these exposure times could be shortened significantly, as we will discuss further.

The primary modification that could potentially reduce the exposure time would be to replace the detector with a state-of-the-art one. The quantum efficiency of the Pilatus 100 K detector that was used in this experiment is only 10% at energies around 30 keV, while that of the recently developed Pilatus3 X CdTe 300 K is 80% at around 30 keV, according to its specifications^27^. Therefore, the exposure time could be reduced by approximately eight times by using a Pilatus3 X CdTe 300 K detector rather than a Pilatus 100 K detector.

The second means of reducing the exposure time would be to use two detectors. In our imaging system, the pinhole collimator and detector system were located on only one side of the incident beam, so the space on the opposite side was free. If two detectors were to be used, the exposure time could be halved. Moreover, if free space existed above or below the sample, additional detectors could be employed.

Thirdly, a multiple-pinhole collimator could be employed, as has been done in SPECT to improve the S/N of projections^28^,^29^. We used a single pinhole in this study; however, the use of multiple pinholes would enable the acquisition of more fluorescent x-ray photons according to the number of pinholes, since the S/N of a projection increases by a factor equal to the number of pinholes. Therefore, if 15 pinholes were used, the exposure time required to obtain projections with the same S/N as that achieved by utilizing a single pinhole would be approximately 15 times shorter.

By incorporating the above improvements, the exposure time could be reduced to about 0.5 s (=2 × 60/8/2/15), which would be sufficiently short even for *in vivo* imaging^18^.

### Dependence on initial value

In general, iterative reconstruction algorithms such as the ML-EM algorithm may depend on the initial values to a greater or lesser extent. Since scatter terms were incorporated into our statistical reconstruction model, unlike in conventional EM-based reconstruction, it is especially important to consider the dependence of the proposed reconstruction algorithm on the initial values of the scatter terms. We compared the reconstruction results obtained using the following three sets of initial conditions: (1) λ_*j*_^(0)^ = 0.0 and *σ*_*i*_^(0)^ = 0.0, (2) λ_*j*_^(0)^ = 0.0 and *σ*_*i*_^(0)^ = 30.0, and (3) λ_*j*_^(0)^ = 0.0 and *σ*_*i*_^(0)^ = 100.0 (1 ≤ *i* ≤ *M*, 1 ≤ *j* ≤ *N*). The reconstructed images were not obviously distinguishable from each other, and the CNR in the 0.1 mg/ml region was 12.1 in each case. Therefore, the reconstruction algorithm exhibited almost no dependence on the initial values of the scatter terms. This result indirectly suggests that the scatter terms effectively and stably suppress the noise caused by scatter components in the reconstruction process.

## Conclusion

We proposed a dual-energy p-FXCT method for scatter correction, in which two sets of projections at incident energies just above and below the K-edge of the imaging agent are obtained and 3-D images are reconstructed from these projections based on a statistical model incorporating the scatter components and ML-EM algorithm. The experimental results demonstrated that dual-energy p-FXCT yields improvements in both image contrast and quality. Although the immediate drawback of this method is the long exposure time, the use of a state-of-the-art detector, an arrangement of multiple detectors, and a multiple-pinhole collimator would enable significant reduction of the required exposure time. The introduction of a multiple-pinhole collimator to increase the FXCT imaging speed will be the focus of our work in the immediate future.

## Additional Information

**How to cite this article:** Sasaya, T. *et al*. Dual-energy fluorescent x-ray computed tomography system with a pinhole design: use of K-edge discontinuity for scatter correction. *Sci. Rep.* **7**, 44143; doi: 10.1038/srep44143 (2017).

**Publisher's note:** Springer Nature remains neutral with regard to jurisdictional claims in published maps and institutional affiliations.

## Supplementary Material

Supplementary Visualization 1

Supplementary Visualization 2

Supplementary Visualization 3

Supplementary Video legend
