# Supplementary material for: Dual-energy fluorescent x-ray computed tomography system with a pinhole design: Use of K-edge discontinuity for scatter correction
Source: Sci Rep. 2017 Mar 8;7:44143. doi: 10.1038/srep44143 (PMC5341157; doi:10.1038/srep44143)
Supplement: Supplementary Video legend [file srep44143-s4.pdf]

Supplementary Information

Paper Title: Dual-energy fluorescent x-ray computed tomography with a pinhole design: use of K-edge discontinuity for scatter correction

Authors: Tenta Sasaya, Naoki Sunaguchi, Thet-Thet-Lwin, Kazuyuki Hyodo, Tsutomu Zeniya, Tohoru Takeda, Tetsuya Yuasa

Video Legends

Visualization 1: Movie of volume rendering of blood vessels in the rat liver reconstructed using dual-energy FXCT.

Visualization 2: Movie of volume rendering of blood vessels in the rat liver reconstructed using mono-energy FXCT.

Visualization 3: Movie of volume rendering of blood vessels in the rat liver reconstructed using attenuation-contrast CT.
